# Supplementary material for: In-situ revitalizing end-of-life MBR membranes via a curtain-type dynamic membrane process
Source: Nat Commun. 2026 Mar 25;17:4383. doi: 10.1038/s41467-026-70969-0 (PMC13181026; doi:10.1038/s41467-026-70969-0)
Supplement: Supplementary file 1 — Supplementary Information [file 41467_2026_70969_MOESM1_ESM.pdf]

SUPPLEMENTARY INFORMATION

In-situ Revitalizing End-of-Life MBR Membranes via a Curtain-Type Dynamic Membrane Process

Yuxiang Liang[1], Yanqing Zhang[2], Fangfang Ye[1], Huan Feng[3], Pingli Li[1], Dongqing Zhan[1], Chenxuan Lou[1], Yangcheng Ding[1], Hai Xiang[1], Xiang Zhang[4], Baojing Gu[5], Fang Liu[6†], Guosheng Shi[7†], Fengchang Wu[8†], Huajun Feng[1†]

1. College of Environment and Resources, College of Carbon Neutral, Zhejiang A&F University, Hangzhou, Zhejiang 311300, China;

2. School of Environmental Science and Engineering, Zhejiang Gongshang University, Hangzhou 310018, Zhejiang, China;

3. Hangzhou Chaoteng Energy Technology Co., Ltd, Hangzhou 310053, Zhejiang, China;

4. College of Energy and Power Engineering, Xihua University, Chengdu, Sichuan 610039, China;

5. College of Environmental and Resource Sciences, Zhejiang University, Hangzhou, China.

6. Zhejiang Province Key Think Tank: Institute of Ecological Civilization and Institute of Carbon Neutrality, Zhejiang A&F University, Hangzhou, China;

7. Shanghai Key Laboratory of Atomic Control and Application of Inorganic 2D Supermaterials, State Key Laboratory of Materials for Advanced Nuclear Energy, Shanghai Applied Radiation Institute, Shanghai University, Shanghai 200444, China;

8. State Key Laboratory of Environmental Criteria and Risk Assessment, Chinese Research Academy of Environmental Sciences, Beijing, 100012, China;

† Corresponding author E-mail address: fenghuajun@zafu.edu.cn

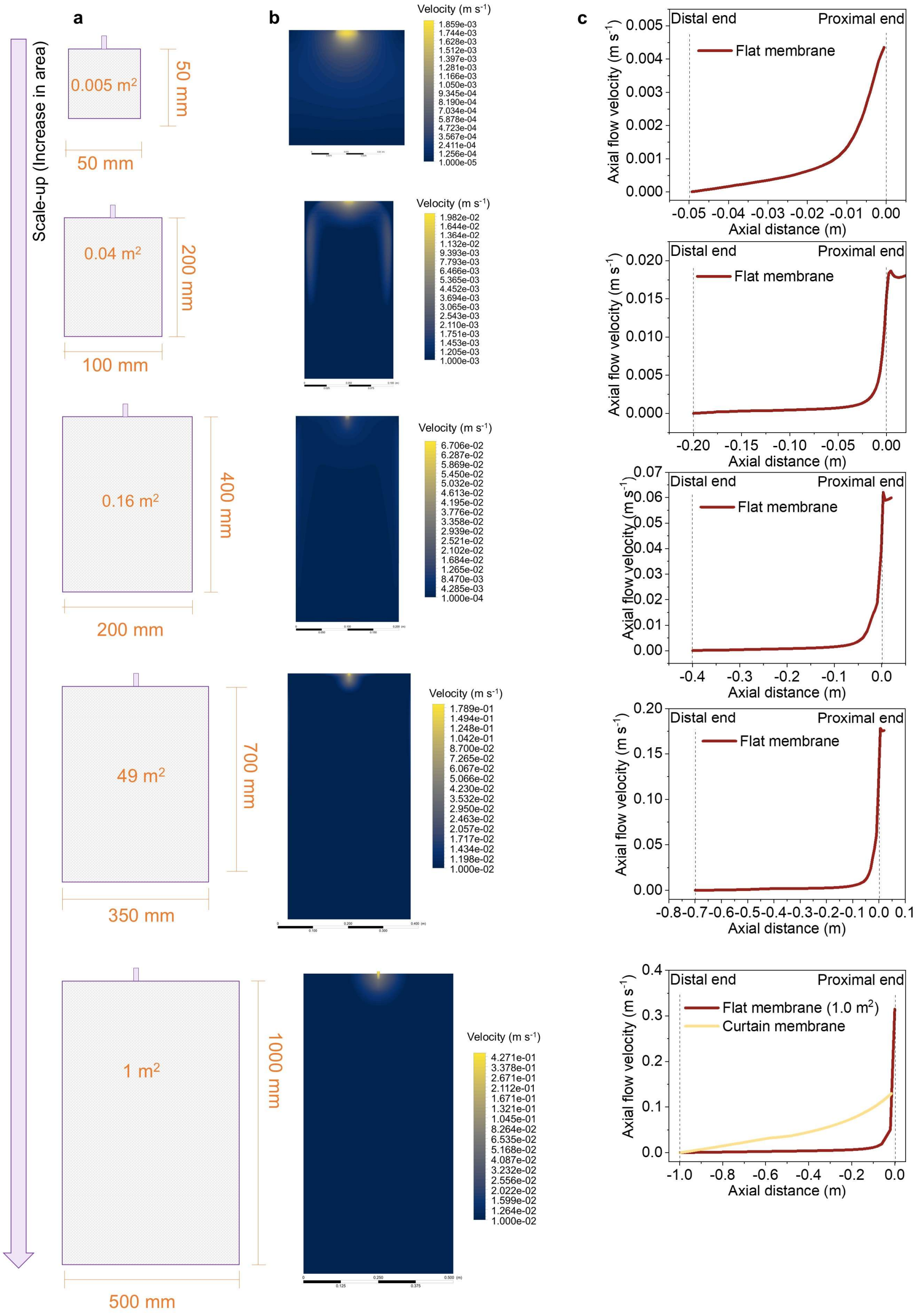

Supplementary Fig. 1 Structural and Hydrodynamic Contrasts of Flat Dynamic Membranes during Scale-Up. (a) Scale-up strategy for flat DM. (b) and (c) Flow velocity distribution for flat DM.

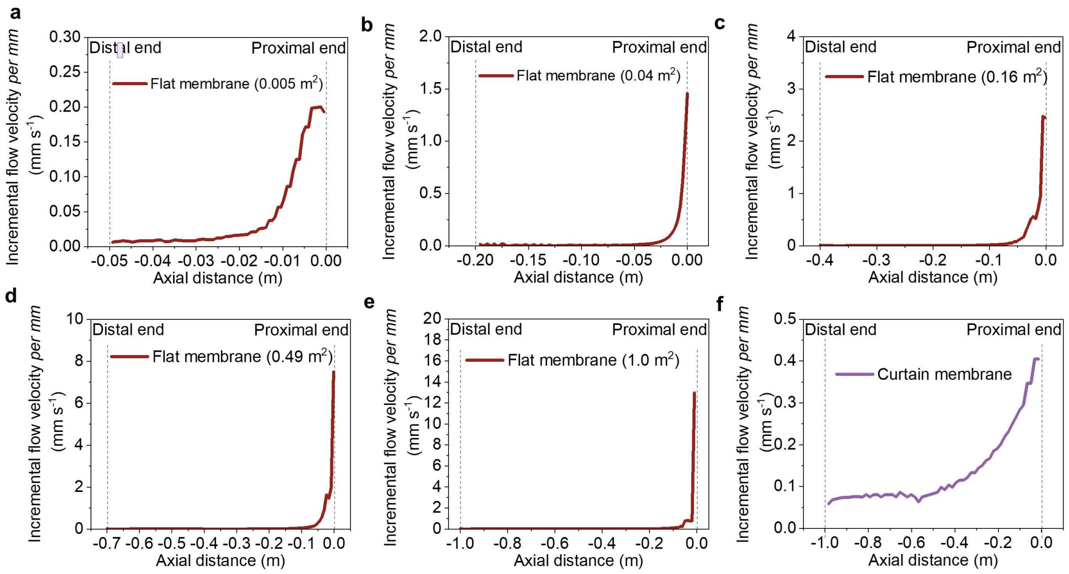

Supplementary Fig. 2 Incremental flow velocity distributions of flat dynamic membranes during scale-up and single membrane filament. (a–e) Incremental flow velocity of flat dynamic membranes with module areas of 0.005, 0.04, 0.16, 0.49, and 1 m^2, respectively. (f) Incremental flow velocity of a single membrane filament.

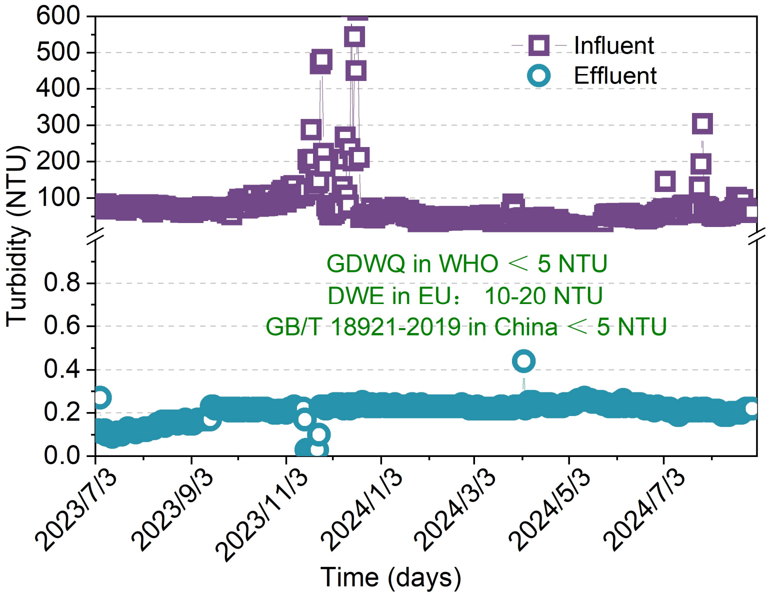

Supplementary Fig. 3 Field study on the turbidity of the effluent from the MBR process with membranes in use for over 6 years.

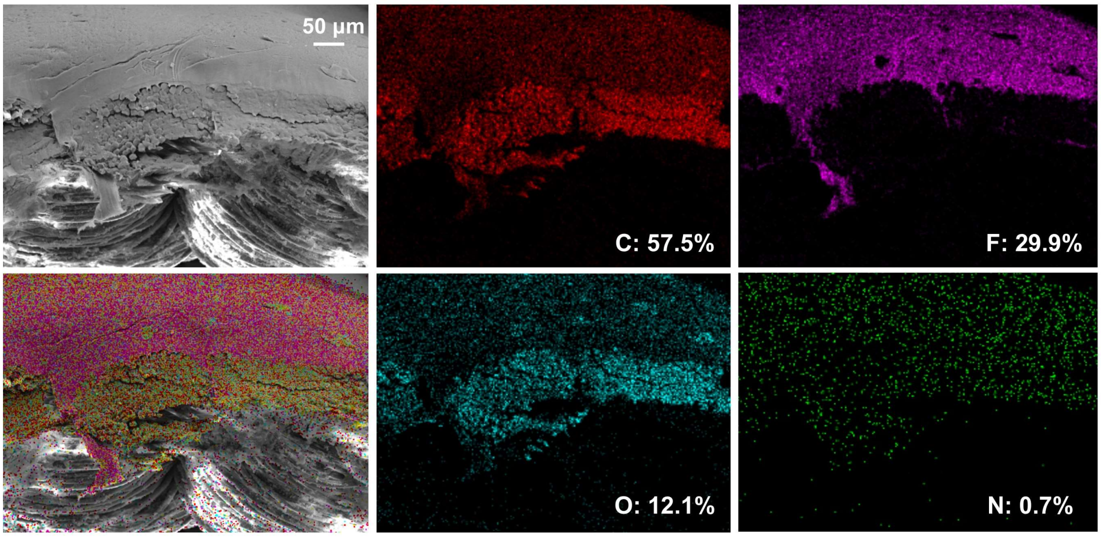

Supplementary Fig. 4 SEM image and EDS elemental characterization of the PVDF membrane cross-section. EDS elemental mapping of C, F, O, N in the cross-section area, with the corresponding elemental content percentages (C: 57.5%, F: 29.9%, O: 12.1%, N: 0.7%) labeled for each element.

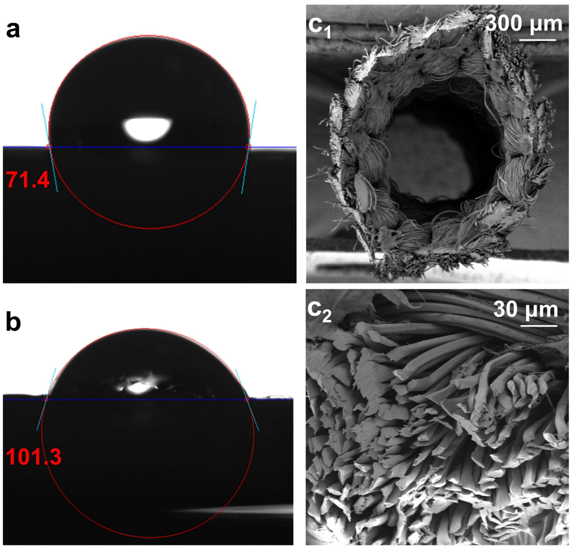

Supplementary Fig. 5 Morphology of internal substrate layer of PVDF membranes. (a) and (b) Contact angles of external PVDF filtration layer and internal substrate layer. (c1) and (c2) SEM images of the internal substrate layer of PVDF membrane.

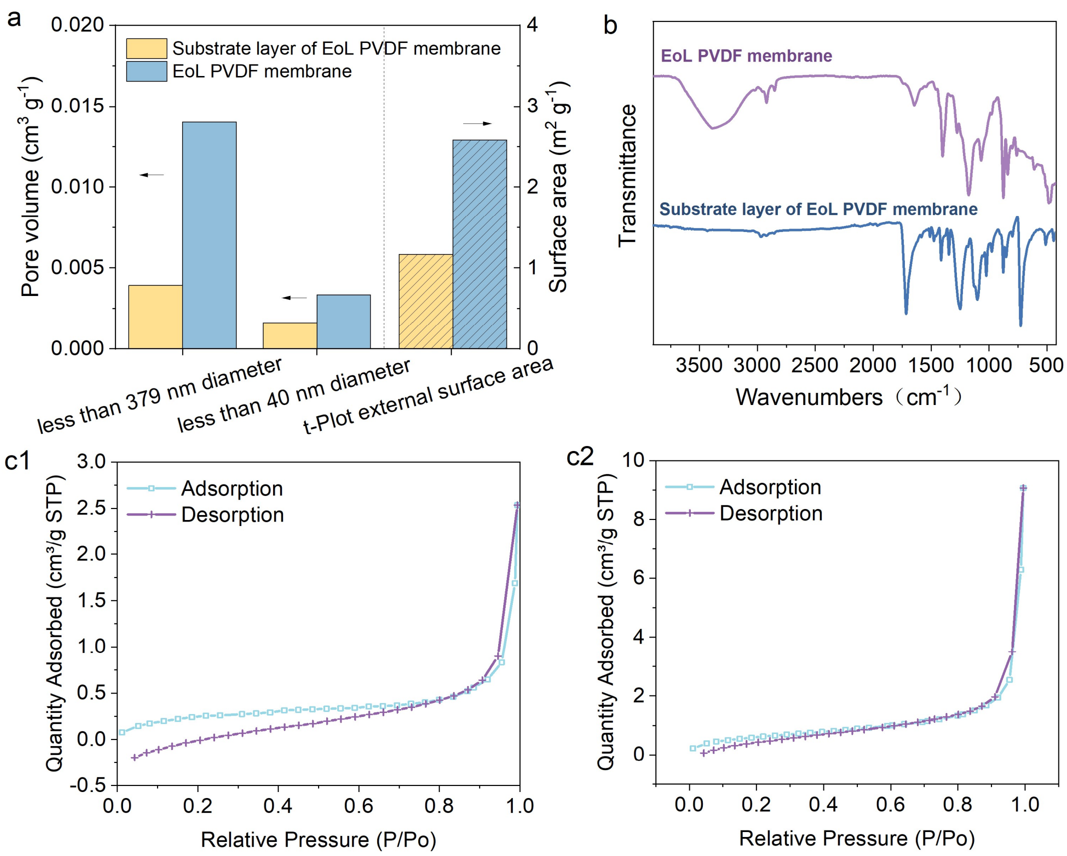

Supplementary Fig. 6. Physicochemical and pore structure characteristics of the PVDF membrane substrate layer. (a) Pore volume and specific surface area differences between the outer PVDF filtration layer (EoL PVDF membrane) and the inner substrate layer (Substrate layer of EoL PVDF membrane). (b) Fourier-transform infrared (FTIR) spectra of the outer PVDF filtration layer and the inner substrate layer. (c1) N_2 adsorption-desorption isotherms (linear plot) of the inner substrate layer. (c2) N_2 adsorption-desorption isotherms (linear plot) of the outer PVDF filtration layer.

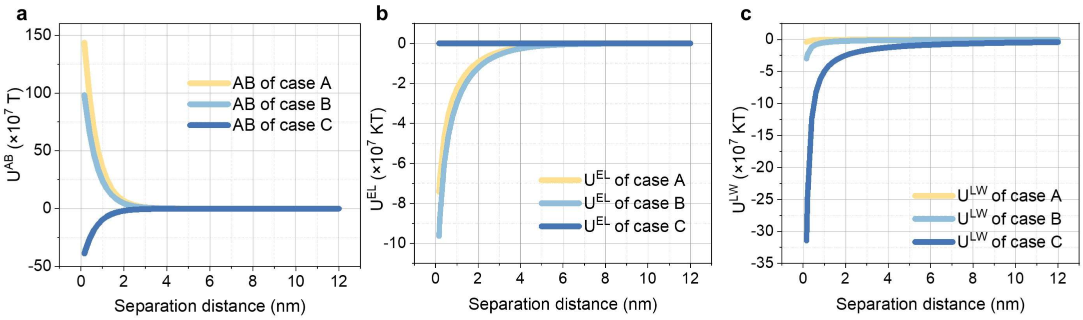

Supplementary Fig. 7 Analysis of the thermodynamic mechanism of membrane surface clogging using the xDLVO theory. (a-c) AB interaction energy, EL interaction energy and LW interaction energy at different stages, respectively.

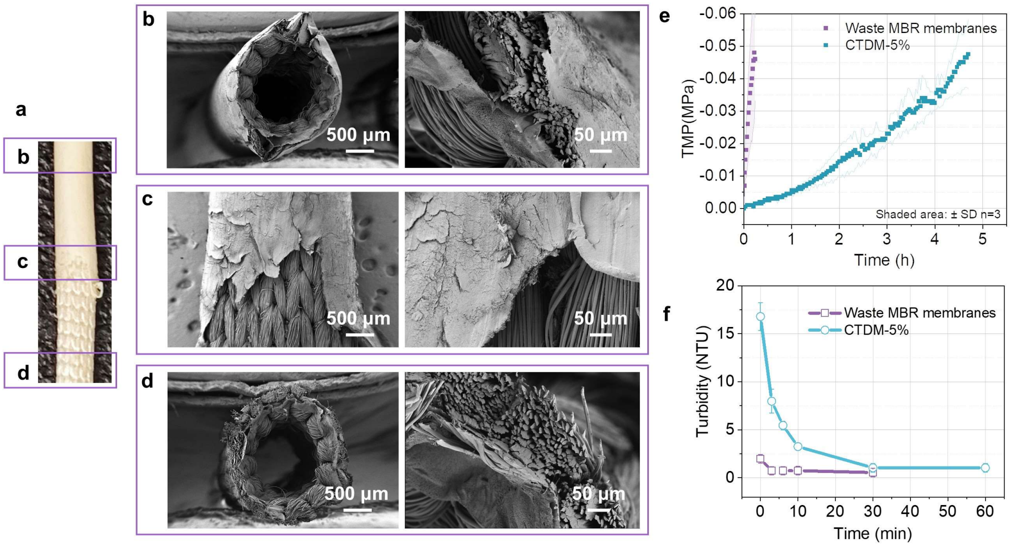

Supplementary Fig. 8 Characteristics of a 5-year-old waste MBR membrane from a municipal wastewater treatment plant (Yuhang District, Hangzhou, Zhejiang, China). (a) Photographic image of the waste MBR membrane. (b) Cross-sectional SEM images of the waste MBR membrane with intact separation layer. (c) Cross-sectional SEM images of the interface between the exposed support layer and intact separation layer. (d) Cross-sectional SEM images of the fully exposed support layer. (e) Transmembrane pressure (TMP) variation of waste MBR membranes vs. CTDM with 5% support layer exposure. (f) Effluent turbidity variation of waste MBR membranes vs. CTDM with 5% support layer exposure.

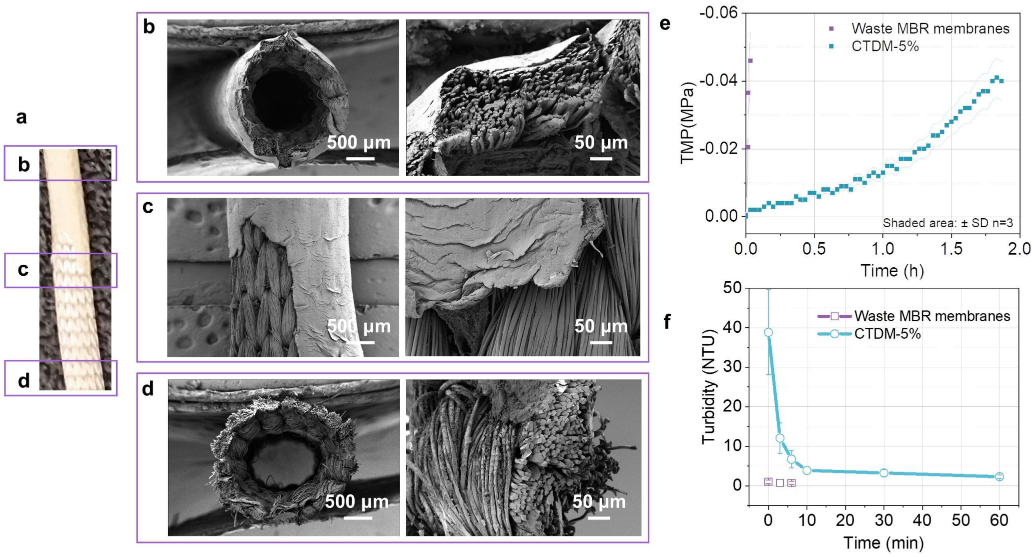

Supplementary Fig. 9 Characteristics of a 5-year-old waste MBR membrane from a livestock wastewater treatment plant (Quzhou, Zhejiang, China). (a) Photographic image of the waste MBR membrane. (b) Cross-sectional SEM images of the waste MBR membrane with intact separation layer. (c) Cross-sectional SEM images of the interface between the exposed support layer and intact separation layer. (d) Cross-sectional SEM images of the fully exposed support layer. (e) Transmembrane pressure (TMP) variation of waste MBR membranes vs. CTDM with 5% support layer exposure. (f) Effluent turbidity variation of waste MBR membranes vs. CTDM with 5% support layer exposure.

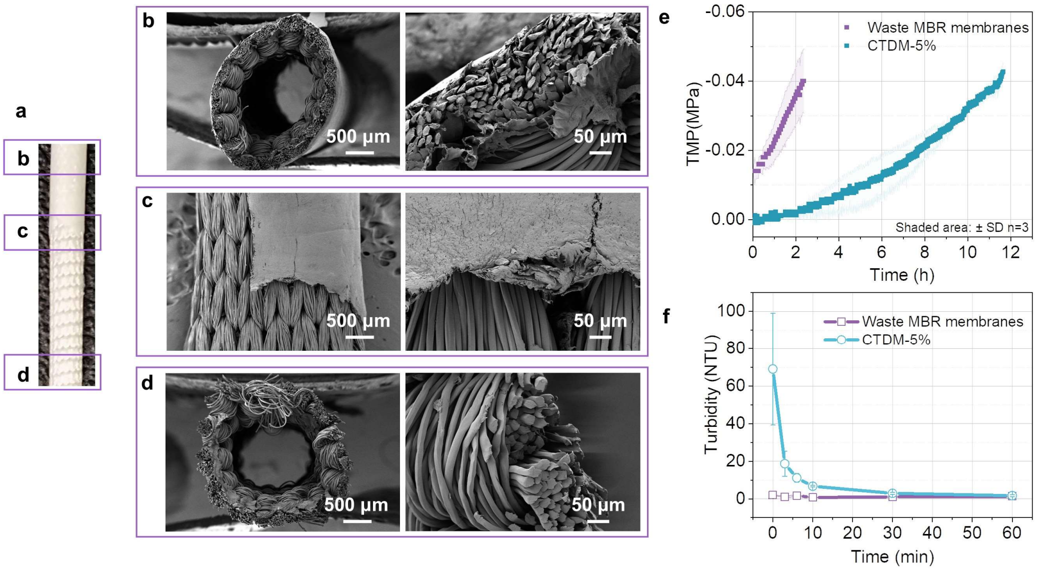

Supplementary Fig. 10 Characteristics of a 6-year-old waste MBR membrane from a rural domestic sewage treatment plant (Taizhou, Zhejiang, China). (a) Photographic image of the waste MBR membrane. (b) Cross-sectional SEM images of the waste MBR membrane with intact separation layer. (c) Cross-sectional SEM images of the interface between the exposed support layer and intact separation layer. (d) Cross-sectional SEM images of the fully exposed support layer. (e) Transmembrane pressure (TMP) variation of waste MBR membranes vs. CTDM with 5% support layer exposure. (f) Effluent turbidity variation of waste MBR membranes vs. CTDM with 5% support layer exposure.

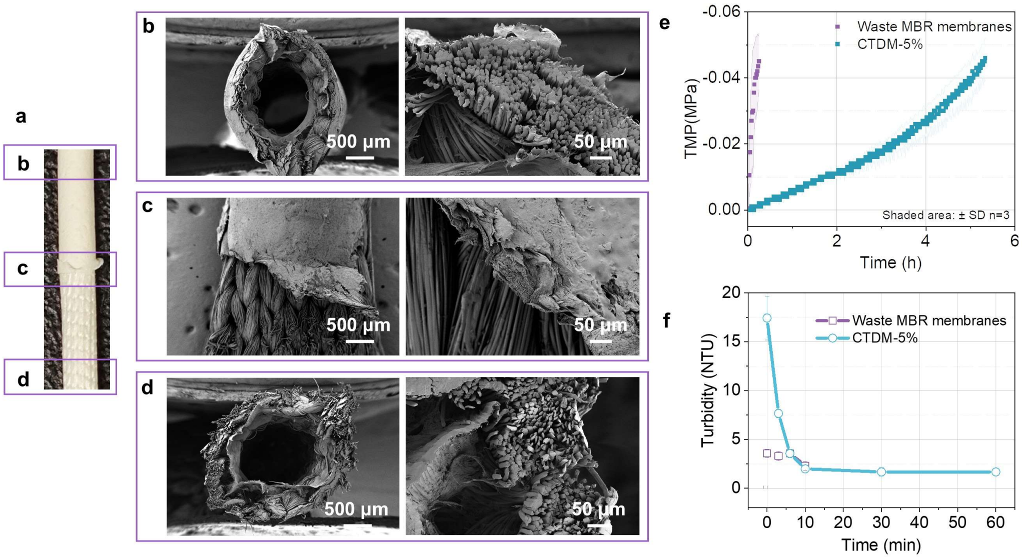

Supplementary Fig. 11 Characteristics of a 5-year-old waste MBR membrane from a plastic cleaning wastewater treatment plant (Ningbo, Zhejiang, China). (a) Photographic image of the waste MBR membrane. (b) Cross-sectional SEM images of the waste MBR membrane with intact separation layer. (c) Cross-sectional SEM images of the interface between the exposed support layer and intact separation layer. (d) Cross-sectional SEM images of the fully exposed support layer. (e) Transmembrane pressure (TMP) variation of waste MBR membranes vs. CTDM with 5% support layer exposure. (f) Effluent turbidity variation of waste MBR membranes vs. CTDM with 5% support layer exposure.

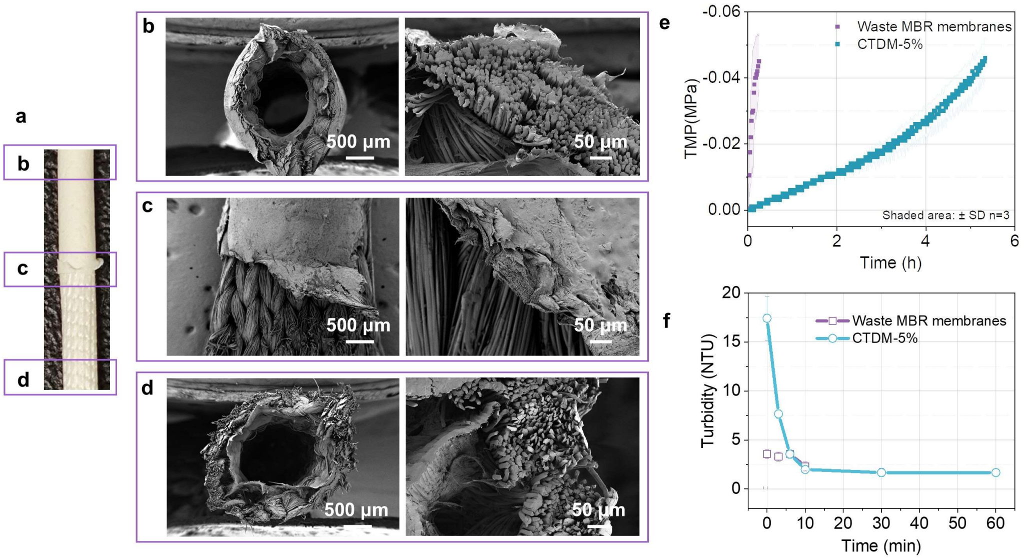

Supplementary Fig. 12 Characteristics of a 5-year-old waste MBR membrane from a printing and dyeing wastewater treatment plant (Shaoxing, Zhejiang, China). (a) Photographic image of the waste MBR membrane. (b) Cross-sectional SEM images of the waste MBR membrane with intact separation layer. (c) Cross-sectional SEM images of the interface between the exposed support layer and intact separation layer. (d) Cross-sectional SEM images of the fully exposed support layer. (e) Transmembrane pressure (TMP) variation of waste MBR membranes vs. CTDM with 5% support layer exposure. (f) Effluent turbidity variation of waste MBR membranes vs. CTDM with 5% support layer exposure.

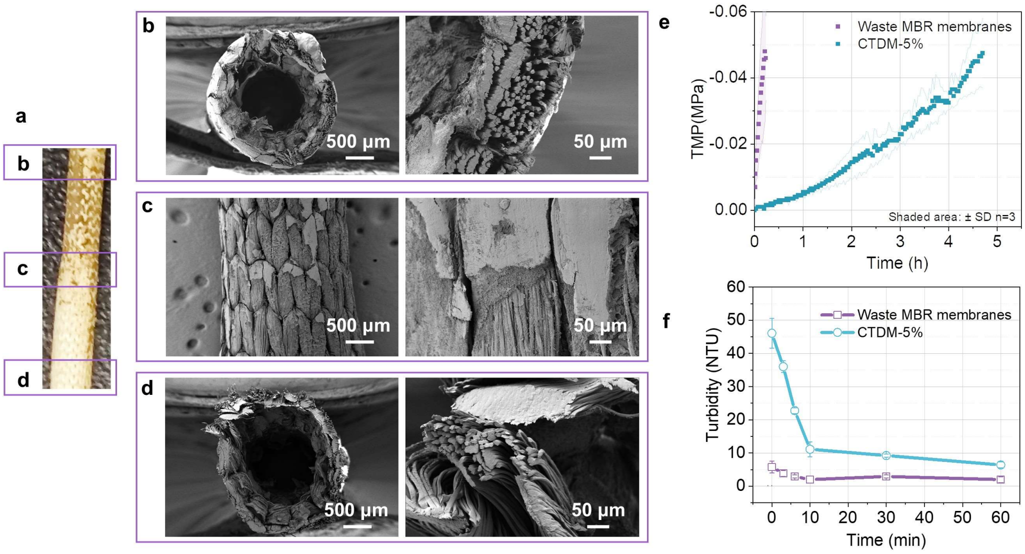

Supplementary Fig. 13 Characteristics of a 4-year-old waste MBR membrane from a pharmaceutical wastewater treatment plant (Taizhou, Zhejiang, China). (a) Photographic image of the waste MBR membrane. (b) Cross-sectional SEM images of the waste MBR membrane with intact separation layer. (c) Cross-sectional SEM images of the interface between the exposed support layer and intact separation layer. (d) Cross-sectional SEM images of the fully exposed support layer. (e) Transmembrane pressure (TMP) variation of waste MBR membranes vs. CTDM with 5% support layer exposure. (f) Effluent turbidity variation of waste MBR membranes vs. CTDM with 5% support layer exposure.

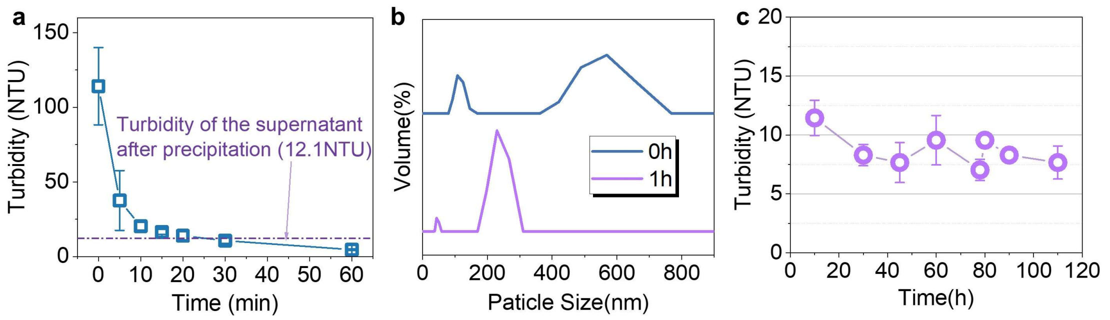

Supplementary Fig. 14 Changes of turbidity and particle size of effluent from 5% exposed internal substrate layer of PVDF membranes during activated sludge treatment of domestic sewage. (a) and (b) Turbidity and effluent particle size change at 0-60 minutes. (c) Turbidity at 60 minutes of every batch.

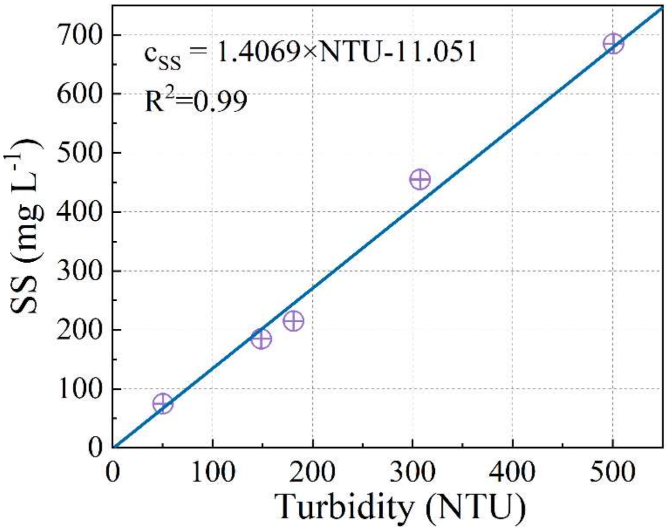

Supplementary Fig. 15 Correlation between turbidity of effluent and SS.

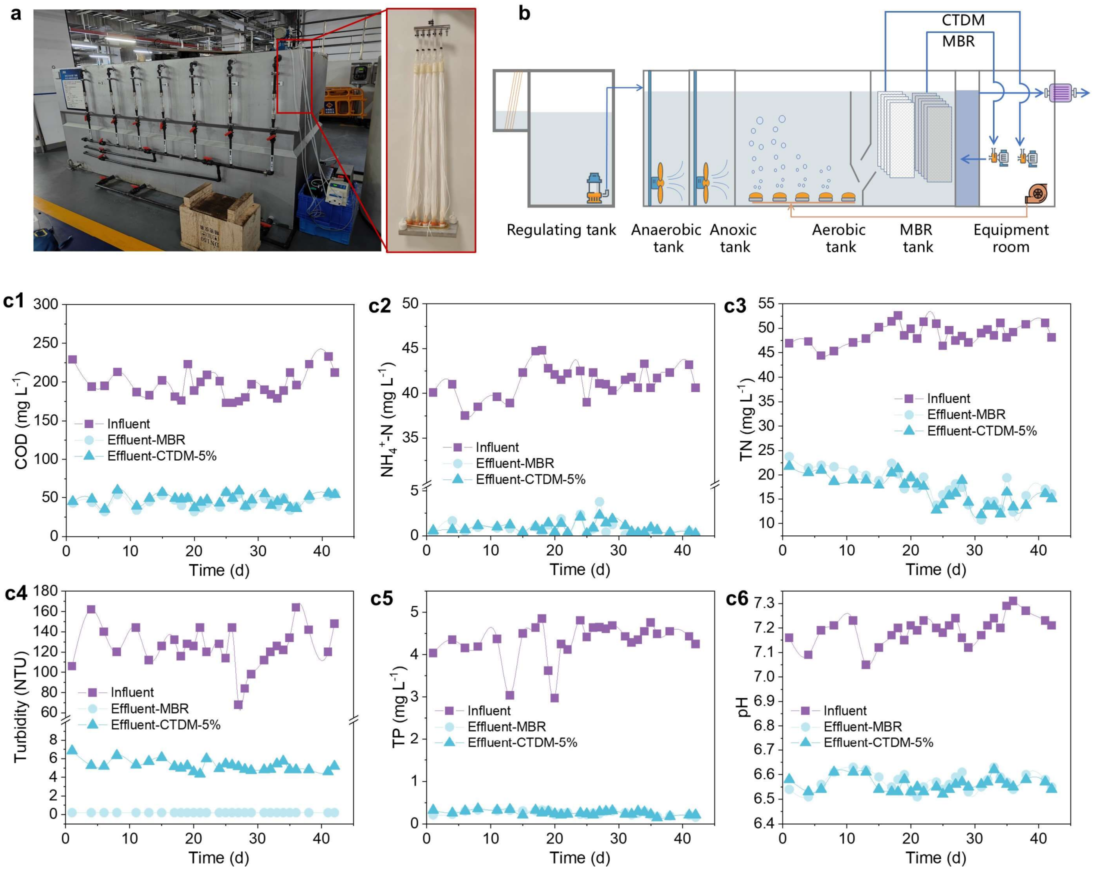

Supplementary Fig. 16 Pilot-scale comparison of effluent quality between MBR and CTDM processes. (a) Photograph of the 20-ton-per-day pilot-scale setup. (b) Flowchart of the integrated wastewater treatment system. (c1-c6) Temporal variations in key water quality parameters (COD, NH_4^+-N, TN, TP, Turbidity, and pH) of the influent, MBR effluent, and CTDM-5% effluent over a 20-day operation.

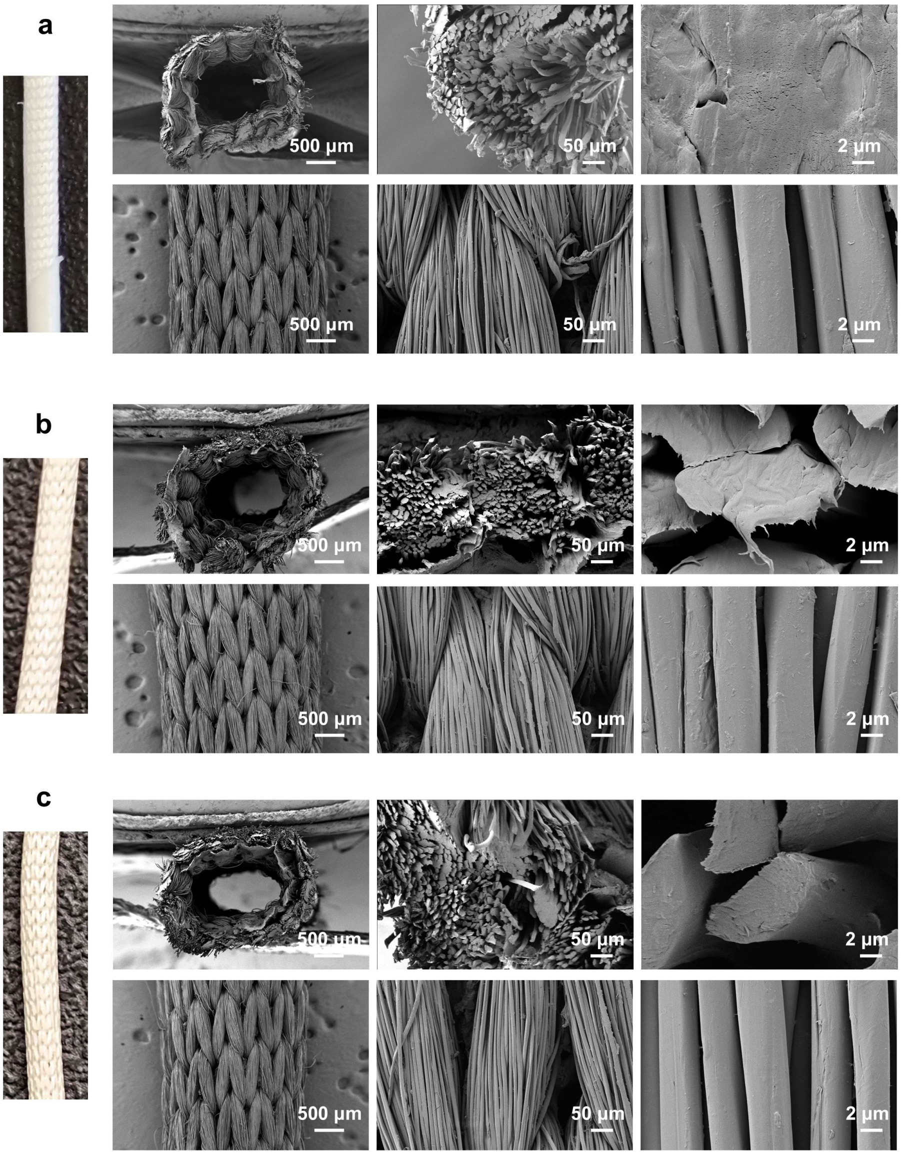

Supplementary Fig. 17 Morphological evolution of exposed support layer of waste MBR membrane during long-term operation. (a-c) Photographic image and SEM images of the support layer immediately after exposure 0 days, 20 days, and 45 days of operation.

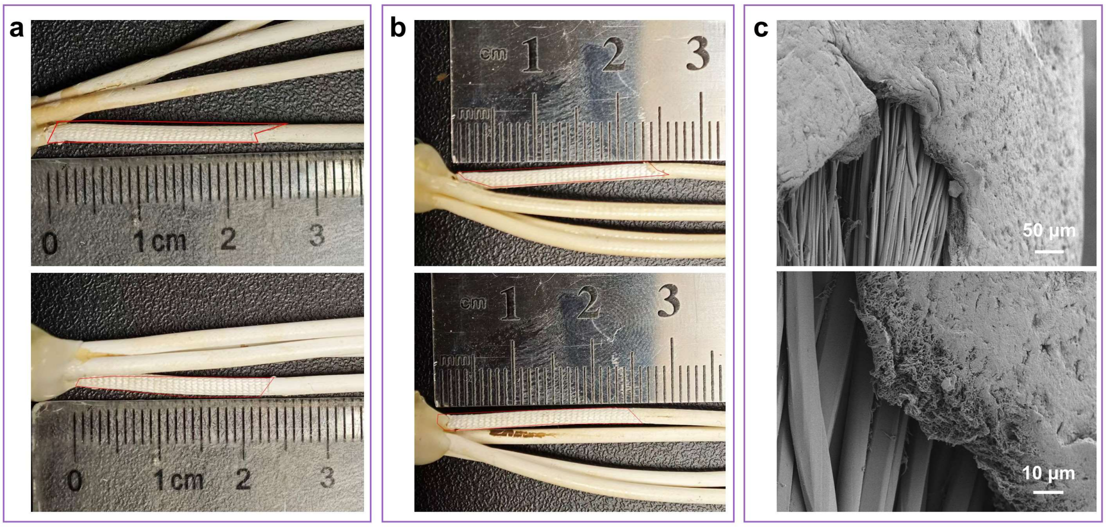

Supplementary Fig. 18 Characterization of the 5% exposed support layer interface of CTDM membrane before and after long-term operation. (a) Dimensional record of the 5% peeled area of the membrane prior to system startup. (b) Dimensional record of the 5% peeled area of the membrane after 45 days of continuous operation. (c) SEM images of the peeled interface of the membrane after 45 days of operation (focusing on the state change of the interface).

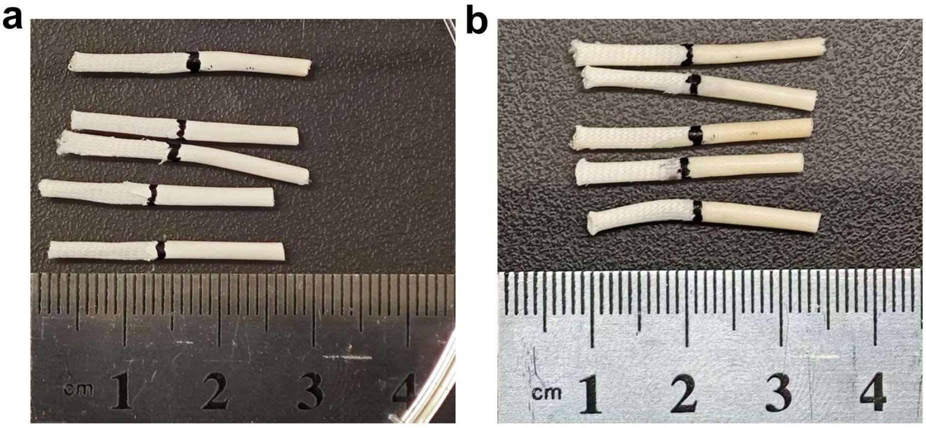

Supplementary Fig. 19 Stability verification of the PVDF layer peeled interface via ultrasonic test. (a) Appearance of the membrane (with black marking line adjacent to the peeled interface) prior to ultrasonic treatment. (b) Appearance of the membrane after 48-hour cyclic ultrasonic treatment (No obvious changes of the black marking line are observed).

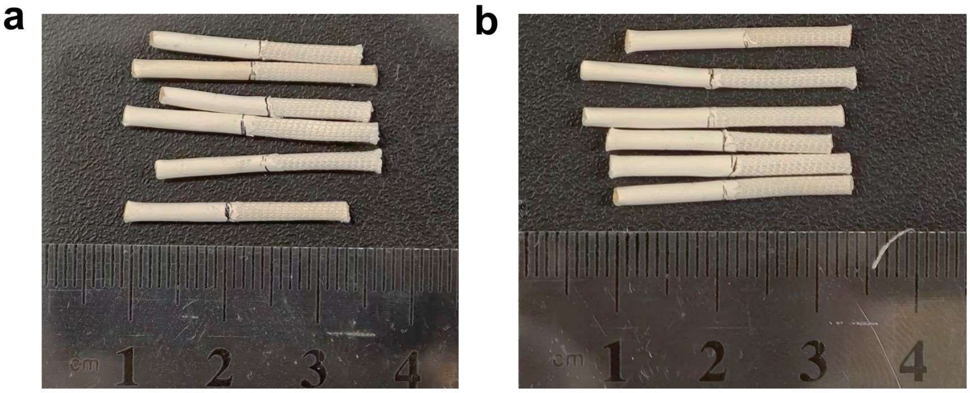

Supplementary Fig. 20 Stability verification of the PVDF layer peeled interface via NaClO immersion test. (a) Appearance of the membrane (with black marking line adjacent to the peeled interface) prior to immersion treatment. (b) Appearance of the membrane after 48-hour immersion treatment (No obvious changes of the black marking line are observed).

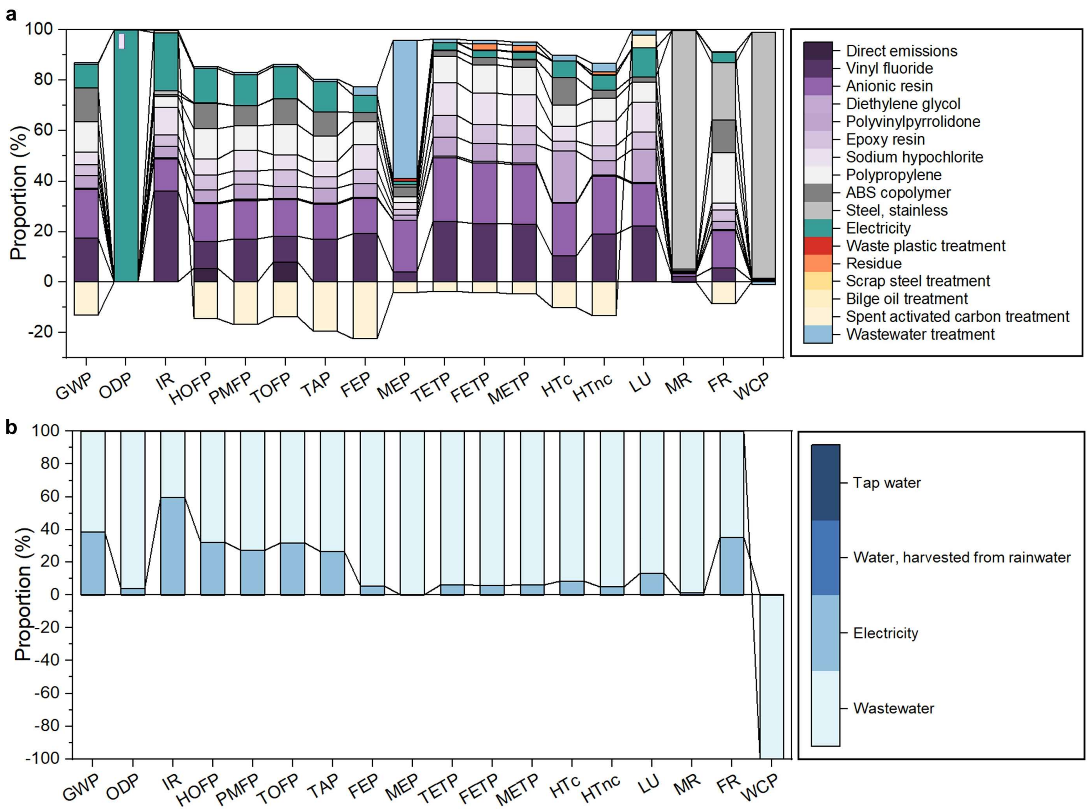

Supplementary Fig. 21 LCA results of option I (a) and option II (b).

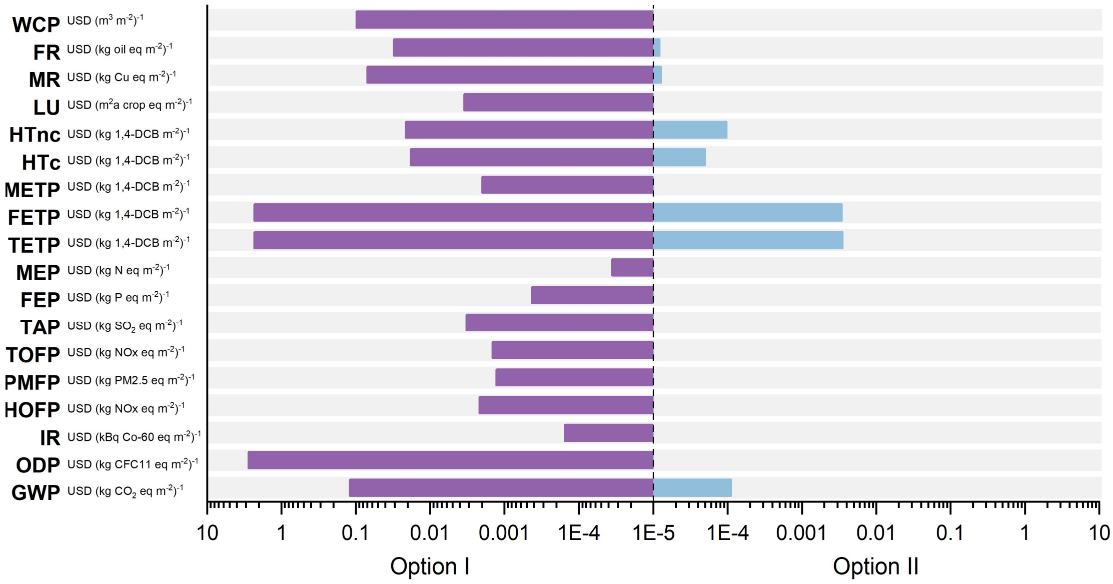

Supplementary Fig. 22 Monetary results of option I and option II.

Supplementary Table 1

Production scale, morphology, and material composition of MBR membranes by company

Company information	scale of MBR membrane (million m^2 a^{-1})	Membranous morphology (%)			Membrane material (%)	
		Flat	Curtain	Other	PVDF	PTFE
Zhejiang Jinmo Environment Technology Co., Ltd.	1-5		80	20	100	
Ningbo Shuiyi Membrane Technology Co., Ltd.	5-10	5	60	35	80	20
Zhejiang Jingyuan Membrane Technology Co., Ltd.	1-5		100		40	60
Hangzhou Gaotong Membrane Technology Co., Ltd.	0.5-1		70	30	100	
Jiangxi Tianyi Aito Membrane Technology Co., Ltd.	1-5	10	90		100	
Zhejiang Kaichuang Environmental Protection Technology Co., Ltd.	5-10		100		100	
Yantai Fengyuan Environmental Protection Equipment Co., Ltd.	1-5	5	95		100	
Hangzhou Kaihong Membrane Technology Co., Ltd.	1-5	10	80	10	100	
Nanjing Rui Jiete Membrane Separation Technology Co., Ltd.	1-5	80	20		100	
Ningbo Jianrong Technology Co., Ltd.	0.5-1		100		100	

Supplementary Table 2

Proportion and advantages of curtain-type membranes with internal substrate layer by company

Company information	Proportion of Curtain-type Membrane with internal substrate layer	Advantages of internal substrate layer			
		Enhanced Filament Strength	High-Intensity Cleaning Requirement	Extended Module Lifespan	Meeting Project Reliability Requirements
Zhejiang Jinmo Environment Technology Co., Ltd.	> 80%	√	√		√
Ningbo Shuiyi Membrane Technology Co., Ltd.	50%-80%	√		√	
Zhejiang Jingyuan Membrane Technology Co., Ltd.	> 95%	√		√	
Hangzhou Gaotong Membrane Technology Co., Ltd.	> 95%	√	√	√	√
Jiangxi Tianyi Aito Membrane Technology Co., Ltd.	> 95%	√	√	√	√
Zhejiang Kaichuang Environmental Protection Technology Co., Ltd.	> 80%	√	√	√	
Yantai Fengyuan Environmental Protection Equipment Co., Ltd.	> 95%	√	√	√	√
Hangzhou Kaihong Membrane Technology Co., Ltd.	> 80%	√	√	√	√
Nanjing Rui Jiete Membrane Separation Technology Co., Ltd.	20%-50%	√	√		
Ningbo Jianrong Technology Co., Ltd.	> 95%	√	√		√

Supplementary Table 3

Proportion of MBR membrane application scenarios by company

Company information	Proportion of MBR Membrane Application Scenarios (%)				
	Municipal Wastewater	Industrial Wastewater	Agricultural Wastewater	Decentralized Rural Sewage	Others (e.g., Drinking Water, Seawater/Brackish Water Desalination, Reclaimed Water)
Zhejiang Jinmo Environment Technology Co., Ltd.	20	75			5
Ningbo Shuiyi Membrane Technology Co., Ltd.	30	50		5	15
Zhejiang Jingyuan Membrane Technology Co., Ltd.	30	50	5	10	5
Hangzhou Gaotong Membrane Technology Co., Ltd.	30	20	5	20	25
Jiangxi Tianyi Aito Membrane Technology Co., Ltd.	40	25	3	15	17
Zhejiang Kaichuang Environmental Protection Technology Co., Ltd.	90	5	5		
Yantai Fengyuan Environmental Protection Equipment Co., Ltd.	20	10	10	40	20
Hangzhou Kaihong Membrane Technology Co., Ltd.	40	40		10	10
Nanjing Rui Jiete Membrane Separation Technology Co., Ltd.	30	50			20
Ningbo Jianrong Technology Co., Ltd.	50	50			

Supplementary Table 4

Consumption and emission inventory of replacing new PVDF membrane (1 m^2 membrane)

Category		Detailed list	Data	
1. land use		land use (m^2 m^{-2})	0.03213	
2. Personnel input		Working time (h m^{-2})	0.96000	
3. Input	3.1 materials	PVDF resin (kg m^{-2})	0.06667	
		DMAC (kg m^{-2})	0.20000	
		PEG (kg m^{-2})	0.00667	
		PVP (kg m^{-2})	0.00667	
		Epoxy resin (kg m^{-2})	0.03333	
		NaClO (kg m^{-2})	0.06667	
		Fiber rope (kg m^{-2})	0.18750	
		ABS plastic case (kg m^{-2})	0.10000	
		Stainless steel frame (kg m^{-2})	0.33333	
	3.2 Water	Water (m^3 m^{-2})	0.03080	
	3.3 energy use	Electricity (kW·h m^{-2})	0.40000	
4. Output	4.1 Emissions to air		before treatment	after treatment
		Smoke (kg m^{-2})	0.00067	0.00005
		NMHC (kg m^{-2})	0.02333	0.00210
	4.2 Emissions to wastewater	Domestic sewage amount (m^3 m^{-2})	0.00430	
		Production wastewater amount (m^3 m^{-2})	0.0234	
			before treatment	after treatment
		COD (kg m^{-2})	0.00702	0.00140
		BOD (kg m^{-2})	0.00351	0.00047
		SS (kg m^{-2})	0.00468	0.00070
	4.3 Emissions to Solid waste	Production waste (kg m^{-2})	0.00333	
		Saturated activated carbon (kg m^{-2})	0.06667	
		Waste material tank (kg m^{-2})	0.00500	
		Waste rag (kg m^{-2})	0.00167	

Supplementary Table 5

Consumption and emission inventory of reusing the end-of-life membrane (1 m^2 membrane)

Category		Detailed list	Data
1. land use		land use (m^2 m^{-2})	0.00000
2. Personnel input		Working time (h m^{-2})	0.00550
3. Input	3.2 Water	Water (m^3 m^{-2})	0.00270
	3.3 energy use	Electricity (kW·h m^{-2})	0.00111
4. Output		Production wastewater amount (m^3 m^{-2})	0.00270

Supplementary Table 6-1

LCA results of replacing new PVDF membrane (1 m^2 membrane)

Impact category	Unit	Total	Direct emissions	PVDF	DMAC	PEG	PVP	ER	NaOCl	PP
**GWP**	kg CO_2 eq	2.6742236	0.0000000	0.6317375	0.7044574	0.0160406	0.1829503	0.1583168	0.1753377	0.4423796
**ODP**	kg CFC11 eq	1.0000427	0.0000000	0.0000001	0.0000423	0.0000000	0.0000001	0.0000000	0.0000002	0.0000001
**IR**	kBq Co-60 eq	0.1485507	0.0000000	0.0538025	0.0187110	0.0005811	0.0070560	0.0065110	0.0162462	0.0063916
**HOFP**	kg NO_x eq	0.0049962	0.0003780	0.0007561	0.0010548	0.0000323	0.0003494	0.0004253	0.0004425	0.0008502
**PMFP**	kg PM2.5 eq	0.0032007	0.0000000	0.0008303	0.0007330	0.0000230	0.0002925	0.0002549	0.0003951	0.0004662
**TOFP**	kg NO_x eq	0.0054808	0.0006090	0.0007699	0.0010973	0.0000340	0.0003598	0.0004983	0.0004471	0.0009068
**TAP**	kg SO_2 eq	0.0070930	0.0000000	0.0019742	0.0016338	0.0000462	0.0006889	0.0005311	0.0006889	0.0011907
**FEP**	kg P eq	0.0004998	0.0000000	0.0001745	0.0001273	0.0000046	0.0000480	0.0000511	0.0000885	0.0000838
**MEP**	kg N eq	0.0002726	0.0000000	0.0000113	0.0000618	0.0000003	0.0000058	0.0000065	0.0000083	0.0000068

**TETP**	kg 1,4-DCB eq	5.7428047	0.0000000	1.4913839	1.5678132	0.0383885	0.4663628	0.5397393	0.8049816	0.6420787
**FETP**	kg 1,4-DCB eq	0.1056627	0.0000000	0.0269113	0.0276199	0.0007872	0.0081587	0.0087903	0.0143272	0.0129212
**METP**	kg 1,4-DCB eq	0.1369461	0.0000000	0.0349864	0.0357036	0.0010147	0.0109372	0.0112569	0.0185183	0.0167663
**HTc**	kg 1,4-DCB eq	0.1145591	0.0000000	0.0150166	0.0298993	0.0005205	0.0293430	0.0055765	0.0084729	0.0118946
**HTnc**	kg 1,4-DCB eq	1.9561258	0.0000000	0.5108158	0.6092586	0.0139510	0.1537336	0.1540231	0.2600002	0.2471255
**LU**	m^2 a crop eq	0.0356231	0.0000000	0.0078926	0.0060220	0.0001775	0.0046677	0.0024209	0.0042025	0.0028612
**MR**	kg Cu eq	0.1601778	0.0000000	0.0034302	0.0017187	0.0000466	0.0003164	0.0006137	0.0008528	0.0010657
**FR**	kg oil eq	1.3205263	0.0000000	0.0877544	0.2394851	0.0084731	0.0504725	0.0719094	0.0432189	0.3192394
**WCP**	m^3	2.3441064	0.0000000	0.0085482	0.0070525	0.0001945	0.0028873	0.0019434	0.0040789	0.0038211

Supplementary Table 6-2

LCA results of replacing new PVDF membrane (1 m^2 membrane)

Impact category	Unit	ABS	SS	Electricity	Waste plastic	Residue	Scrap steel	Bilge oil	SAC	Waste water
**GWP**	kg CO_2 eq	0.4795204	0.0000008	0.3446482	0.0001925	0.0000674	0.0000261	0.0000393	-0.4772421	0.0157513
**ODP**	kg CFC11 eq	0.0000000	0.0000000	1.0000000	0.0000000	0.0000000	0.0000000	0.0000000	-0.0000001	0.0000000
**IR**	kBq Co-60 eq	0.0008345	0.0025430	0.0341301	0.0000007	0.0000013	0.0000007	0.0000001	0.0010793	0.0006616
**HOFP**	kg NO_x eq	0.0007029	0.0000132	0.0009603	0.0000001	0.0000003	0.0000002	0.0000000	-0.0010277	0.0000582
**PMFP**	kg PM2.5 eq	0.0003829	0.0000013	0.0005929	0.0000001	0.0000002	0.0000001	0.0000001	-0.0008172	0.0000454
**TOFP**	kg NO_x eq	0.0007614	0.0000139	0.0009623	0.0000001	0.0000003	0.0000002	0.0000000	-0.0010388	0.0000592
**TAP**	kg SO_2 eq	0.0011067	0.0000044	0.0013899	0.0000001	0.0000005	0.0000002	0.0000001	-0.0022735	0.0001109
**FEP**	kg P eq	0.0000332	0.0000000	0.0000618	0.0000000	0.0000000	0.0000000	0.0000000	-0.0002042	0.0000312
**MEP**	kg N eq	0.0000114	0.0000025	0.0000046	0.0000030	0.0000000	0.0000000	0.0000000	-0.0000126	0.0001629

**TETP**	kg 1,4-DCB eq	0.1459696	0.0028505	0.1891320	0.0000624	0.0001273	0.0000891	0.0000343	-0.2303426	0.0841341
**FETP**	kg 1,4-DCB eq	0.0033420	0.0000219	0.0032064	0.0002626	0.0027467	0.0000005	0.0000006	-0.0049467	0.0015130
**METP**	kg 1,4-DCB eq	0.0045540	0.0000225	0.0044607	0.0003528	0.0037243	0.0000007	0.0000008	-0.0073378	0.0019846
**HTc**	kg 1,4-DCB eq	0.0157410	0.0000003	0.0093493	0.0000052	0.0003623	0.0000007	0.0000009	-0.0145935	0.0029695
**HTnc**	kg 1,4-DCB eq	0.0829652	0.0001097	0.1553723	0.0068575	0.0319051	0.0000106	0.0000250	-0.3569394	0.0869119
**LU**	m^2 a crop eq	0.0007334	0.0000000	0.0040655	0.0000049	0.0000097	0.0000042	0.0000008	0.0017794	0.0007811
**MR**	kg Cu eq	0.0001788	0.1516609	0.0001419	0.0000001	0.0000001	0.0000001	0.0000000	-0.0001426	0.0002945
**FR**	kg oil eq	0.2064367	0.3618316	0.0664737	0.0000097	0.0000189	0.0000169	0.0000064	-0.1383106	0.0034903
**WCP**	m^3	0.0058146	2.3338585	0.0011782	0.0000004	0.0000009	0.0000008	0.0000000	-0.0004739	-0.0247991

Supplementary Table 7

LCA results of reusing the end-of-life membrane (1 m^2 membrane)

Impact category	Unit	Total	Tap water	Water, harvested from rainwater	Electricity,	Wastewater, average
**GWP**	kg CO_2 eq	0.00249335	0.00000097	0.00000066	0.00095640	0.00153533
**ODP**	kg CFC11 eq	0.00000000	0.00000000	0.00000000	0.00000000	0.00000000
**IR**	kBq Co-60 eq	0.00015937	0.00000016	0.00000002	0.00009471	0.00006448
**HOFP**	kg NO_x eq	0.00000835	0.00000000	0.00000000	0.00000266	0.00000568
**PMFP**	kg PM2.5 eq	0.00000607	0.00000000	0.00000000	0.00000165	0.00000442
**TOFP**	kg NO_x eq	0.00000844	0.00000000	0.00000000	0.00000267	0.00000577
**TAP**	kg SO_2 eq	0.00001467	0.00000000	0.00000000	0.00000386	0.00001081
**FEP**	kg P eq	0.00000321	0.00000000	0.00000000	0.00000017	0.00000304
**MEP**	kg N eq	0.00001589	0.00000000	0.00000000	0.00000001	0.00001588

**TETP**	kg 1,4-DCB	0.00873042	0.00000187	0.00000292	0.00052484	0.00820080
**FETP**	kg 1,4-DCB	0.00015646	0.00000005	0.00000004	0.00000890	0.00014748
**METP**	kg 1,4-DCB	0.00020593	0.00000006	0.00000005	0.00001238	0.00019344
**HTc**	kg 1,4-DCB	0.00031558	0.00000008	0.00000011	0.00002594	0.00028944
**HTnc**	kg 1,4-DCB	0.00890451	0.00000113	0.00000066	0.00043116	0.00847156
**LU**	m^2 a crop eq	0.00008769	0.00000002	0.00000025	0.00001128	0.00007614
**MR**	kg Cu eq	0.00002912	0.00000001	0.00000001	0.00000039	0.00002871
**FR**	kg oil eq	0.00052506	0.00000026	0.00000013	0.00018446	0.00034021
**WCP**	m^3	-0.00241125	0.00000272	0.00000001	0.00000327	-0.00241724

Supplementary Table 8

Monetary result of replacing new PVDF membrane (1 m^2 membrane)

Impact category	LCA result for 1 m^2 membrane	Unit	Monetary factor	Unit	Monetary result for 1 m^2 membrane
**GWP**	2.67422364	kg CO_2 eq	0.32624851	USD kg^{-1} CO_2 eq	0.12013774
**ODP**	1.00004273	kg CFC11 eq	20.22267604	USD kg^{-1} CFC11 eq	2.78478146
**IR**	0.14855065	kBq Co-60 eq	0.00754895	USD kBq^{-1} Co-60 eq	0.00015422
**HOFP**	0.00499615	kg NO_x eq	3.14773433	USD kg^{-1} NO_x eq	0.00216602
**PMFP**	0.00320072	kg PM2.5 eq	2.92570117	USD kg^{-1} PM2.5 eq	0.00128887
**TOFP**	0.00548075	kg NO_x eq	1.92225195	USD kg^{-1} NO_x eq	0.00145136
**TAP**	0.00709299	kg SO_2 eq	3.33509077	USD kg^{-1} SO_2 eq	0.00325798
**FEP**	0.00049980	kg P eq	6.15801605	USD kg^{-1} P eq	0.00042412
**MEP**	0.00027263	kg N eq	0.95146960	USD kg^{-1} N eq	0.00003572

**TETP**	5.74280472	kg 1,4-DCB	2.93521334	USD kg^{-1} 1,4-DCB	2.32112077
**FETP**	0.10566273	kg 1,4-DCB	160.00000000	USD kg^{-1} 1,4-DCB	2.32796171
**METP**	0.13694605	kg 1,4-DCB	0.10600432	USD kg^{-1} 1,4-DCB	0.00199940
**HTc**	0.11455911	kg 1,4-DCB	1.15164105	USD kg^{-1} 1,4-DCB	0.01816676
**HTnc**	1.95612575	kg 1,4-DCB	0.07908860	USD kg^{-1} 1,4-DCB	0.02130357
**LU**	0.03562314	m^2 a crop eq	0.70780831	USD m^{-2} a crop eq	0.00347142
**MR**	0.16017783	kg Cu eq	3.16878928	USD kg^{-1} Cu eq	0.06989239
**FR**	1.32052630	kg oil eq	0.16850669	USD kg^{-1} oil eq	0.03064100
**WCP**	2.34410639	m^3	0.30322581	USD m^{-3}	0.09787578
**Total**					

Supplementary Table 9

Monetary result of reusing the end-of-life membrane (1 m^2 membrane)

**Impact category**	**LCA result for 1 m^2 membrane**	**Unit**	**Monetary factor**	**Unit**	**Monetary result for 1 m^2 membrane**
**GWP**	0.00249335	kg CO_2 eq	0.32624851	USD kg^{-1} CO_2 eq	0.00011201
**ODP**	0.00000000	kg CFC11 eq	20.22267604	USD kg^{-1} CFC11 eq	0.00000001
**IR**	0.00015937	kBq Co-60 eq	0.00754895	USD kBq^{-1} Co-60 eq	0.00000017
**HOFP**	0.00000835	kg NO_x eq	3.14773433	USD kg^{-1} NO_x eq	0.00000362
**PMFP**	0.00000607	kg PM2.5 eq	2.92570117	USD kg^{-1} PM2.5 eq	0.00000245
**TOFP**	0.00000844	kg NO_x eq	1.92225195	USD kg^{-1} NO_x eq	0.00000223
**TAP**	0.00001467	kg SO_2 eq	3.33509077	USD kg^{-1} SO_2 eq	0.00000674
**FEP**	0.00000321	kg P eq	6.15801605	USD kg^{-1} P eq	0.00000273
**MEP**	0.00001589	kg N eq	0.95146960	USD kg^{-1} N eq	0.00000208

**TETP**	0.00873042	kg 1,4-DCB	2.93521334	USD kg^{-1} 1,4-DCB	0.00352925
**FETP**	0.00015646	kg 1,4-DCB	160.00000000	USD kg^{-1} 1,4-DCB	0.00344663
**METP**	0.00020593	kg 1,4-DCB	0.10600432	USD kg^{-1} 1,4-DCB	0.00000301
**HTc**	0.00031558	kg 1,4-DCB	1.15164105	USD kg^{-1} 1,4-DCB	0.00005004
**HTnc**	0.00890451	kg 1,4-DCB	0.07908860	USD kg^{-1} 1,4-DCB	0.00009698
**LU**	0.00008769	m^2 a crop eq	0.70780831	USD m^{-2} a crop eq	0.00000855
**MR**	0.00002912	kg Cu eq	3.16878928	USD kg^{-1} Cu eq	0.00001271
**FR**	0.00052506	kg oil eq	0.16850669	USD kg^{-1} oil eq	0.00001218
**WCP**	-0.00241125	m^3	0.30322581	USD m^{-3}	-0.00010068
**Total**					0.00719071

Supplementary Table 10

Computational parameters related to fluid dynamics

**Project**	**Parameter**
Analysis type	Steady state
Turbulence model	The Baseline (BSL) k-omega Model
Wall function	Automatic
Numerical accuracy of convective term	High resolution (2nd order)
Numerical accuracy of turbulence term	High resolution (2nd order)
Gravitational acceleration g	9.81 m s^{-2}
Density	997 kg m^{-3}
Dynamic viscosity	8.899×10^{-4} Pa·s
Permeability of porous medium	4.5×10-15 m^2

Supplementary Table 11 The values of each item of equations (1) and (2)

**Project**	**Value**
β'	0.09
α_1	5/9
β_1	0.075
β_2	0.0828
σ_{k1}	2
σ_{k1}	1
σ_2	0.44
σ_{w1}	2
σ_{w2}	1/0.856

Supplementary Table 12

Four classical filtering model formulas and parameters

Fouling mechanism	Model	Blocking constant
Complete blocking	$J = J_0 e^{-K_b t}$	K_b
Standard blocking	$J = \frac{J_0}{\left(1 + \frac{K_s J_0^{\frac{1}{2}} t}{2}\right)^2}$	K_s
Intermediate blocking	$J = \frac{J_0}{(1 + K_i J_0 t)}$	K_i
Cake filtration	$J = \frac{J_0}{(1 + 2K_c J_0^2 t)^{\frac{1}{2}}}$	K_c

Supplementary Table 13

The contact angle, surface tension, grain diameter and Zeta potential of different membranes surface and active sludge.

	Contact angle (º)			Surface tension (mJ m^{-2})			Other indicators	
	water	glycerol	n-hexadecane	y^{LW} (water)	y^{+} (glycerol)	y^{-} (n-hexadecane)	Diameter (nm)	Zeta potential （mV）
New PVDF membranes	90.00	75.31	41.04	20.77	1.85	2.67		-19.30
PVDF membranes used more than 6 years	101.31	82.88	35.54	22.2	1.02	0.18		-33.51
Internal substrate layer	69.45	26.14	23.16	24.87	14.76	1.65		-37.96
Active sludge				64.08	52.08	34.64	59460.00	-1.12

Supplementary Table 14

The interaction free energy between different membranes surface and active sludge.

	Interaction energy per unit area (mJ m^{-2})			
	$\Delta G_{h_0}^{LW}$	$\Delta G_{h_0}^{AB}$	$\Delta G_{h_0}^{EL}$	$\Delta G_{h_0}^{TOT}$
Between new PVDF membranes and active sludge	0.74482	20.97081	-0.68788	21.02775
Between PVDF membranes used more than 6 years and active sludge	-0.28449	26.79901	-2.19040	24.32413
Between internal substrate layer and active sludge	-2.12124	18.33686	-2.83496	13.38066
Between sludge cake and active sludge	-22.25714	-7.24456	0.00028	-29.50143

Supplementary Table 15

Comparison of prediction methods for MBR membrane usage scale in China

Method	Sort	RMSE	MAD	MAPE	Theil's U	Durbin-Watson	Alpha	Beta
**Double exponential smoothing method**	**1**	**0.74**	**0.54**	**3.41%**	**0.5046**	**1.7095**	**0.8474**	**0.9990**
Damped trend non-seasonal	2	0.74	0.54	3.41%	0.5048	1.708	0.8483	0.9990
ARIMA(0,2,0)	3	0.78	0.55	3.26%	0.4122	2.3994		
Second moving average method	4	0.90	0.60	3.29%	0.4317	1.2749		
First moving average method	5	2.93	2.67	18.19%	1.00	0.0644		
Single exponential smoothing method	6	2.93	2.67	18.21%	1.0007	0.0642	0.9990	

Supplementary Table 16

Comparison of prediction methods for MBR sewage scale in China

Method	Sort	RMSE	MAD	MAPE	Theil's U	Durbin-Watson	Alpha	Beta
**ARIMA(2,2,2)**	**1**	**1.64**	**1.22**	**5.24%**	**0.7106**	**1.5173**		
Double exponential smoothing method	2	2.95	2.14	10.13%	0.8562	1.8013	0.7316	0.7320
Damped trend non-seasonal	3	2.95	2.14	10.13%	0.8562	1.7998	0.7303	0.7354
Second moving average method	4	3.59	2.76	10.94%	0.7733	2.1402		
First moving average method	5	5.54	4.51	23.18%	1.00	0.3944		
Single exponential smoothing method	6	5.55	4.51	23.20%	1.0002	0.3936	0.9990	

Supplementary Methods

1. Filtration Performance Test of End-of-Life (EoL) MBR Membranes from Diverse Sources

The experimental reactor was a plastic reactor with dimensions of 14.0×9.0×19.0 cm and an effective volume of 1.5 L. An aeration head was installed at the bottom of the reactor, and air was supplied by an aeration pump (SOBO air pump, SB–988) to provide aeration and mixing. The activated sludge operated in a sequencing batch mode with a hydraulic retention time (HRT) of 6 hours. The system maintained continuous bottom aeration with an air-water ratio of 8, which not only provided dissolved oxygen for microbial metabolism but also mixed the reactor contents and controlled the formation of sludge cake on the membrane surface. Two peristaltic pumps were configured: one for extracting the permeate of the CTDM system, and the other for feeding wastewater at the same flow rate to keep the liquid level in the reactor constant. A pressure transmitter (Asmik MIK-P300) was installed on the effluent pipeline to real-time monitor the transmembrane pressure difference (TMP), which was used to reflect the membrane fouling degree. When the TMP of the membrane module reached -0.04 MPa, it was defined as complete fouling.

The activated sludge was sourced from the aeration tank of Tangqi Wastewater Treatment Plant in in Hangzhou City, Zhejiang Province, China. The concentration of the added sludge was 3500 ± 500 mg L^{-1}, and the sludge SV30 was 20%. Simulated domestic sewage: Sucrose (AR) was used to prepare a COD concentration of 200 mg L^{-1}, NH_4Cl (AR) was used to prepare an ammonia nitrogen concentration of 40 mg L^{-1}, KH_2PO_4 (AR) was used to prepare a total phosphorus concentration of 5 mg L^{-1}, and trace elements (1 mL L^{-1}) were added, with a total volume of 1 L. The trace elements included H_3BO_4 (150 mg L^{-1}), $ZnSO_4{\cdot}7H_2O$ (120 mg L^{-1}), $MnCl_2{\cdot}7H_2O$ (120 mg L^{-1}),

$CuSO_4 \cdot 5H_2O$ (30 mg L^{-1}), Na_2MoO_4 (65 mg L^{-1}), $NiCl_2$ (50 mg L^{-1}), $CoCl_2 \cdot 6H_2O$ (210 mg L^{-1}), and KI (30 mg L^{-1}). All trace element reagents were of AR. The actual wastewater was collected from the influent of the fourth phase of Yuhang Wastewater Treatment Plant in Hangzhou City, Zhejiang Province, China. The system operated at room temperature (22 ± 3°C) throughout the experiment.

The core filtration unit adopted EoL PVDF hollow fiber MBR membranes from various industrial and non-municipal wastewater treatment scenarios. Prior to assembly, all EoL membranes were subjected to unified pretreatment: soaked in 10% citric acid solution for 12 hours and 15% sodium hypochlorite solution for 12 hours sequentially to remove surface adherent impurities (e.g., sludge cake, residual pollutants). For all EoL membrane samples, the PVDF separation layer was artificially peeled off using adhesive tape to achieve a uniform exposed area of 5% (the target exposure ratio in the main study). Six types of EoL PVDF hollow fiber MBR membranes covering typical non-municipal application scenarios were collected, with detailed information as follows:

- Municipal wastewater treatment: Collected from the Phase IV Facility of Yuhang Wastewater Treatment Plant in Hangzhou, Zhejiang Province, China, with a service life of 5 years.
- Livestock wastewater treatment: Collected from a swine farm wastewater treatment plant in Xiaonanhai Town, Quzhou, Zhejiang Province, China, with a service life of 5 years.
- Rural domestic sewage treatment: Collected from a domestic sewage treatment plant in Binhai Town, Taizhou, Zhejiang Province, China, with a service life of 6 years;

- Plastic cleaning wastewater treatment: Collected from Zhejiang Jingyuan Membrane Technology Co., Ltd. in Ningbo, Zhejiang Province, China, with a service life of 5 years;
- Printing and dyeing wastewater treatment: Collected from Shaoxing Shengxin Printing & Dyeing Co., Ltd. in Shaoxing, Zhejiang Province, China, with a service life of 5 years;
- Pharmaceutical wastewater treatment: Collected from a pharmaceutical factory in Jiaojiang District, Taizhou, Zhejiang Province, China, with a service life of 4 years;

2. Pilot-Scale Experimental Setup and Operation

The pilot-scale system was deployed at the Phase IV Facility of Yuhang Wastewater Treatment Plant (Hangzhou, Zhejiang, China), with a designed treatment capacity of 20 t d^{-1}. The unit processed actual municipal wastewater from the plant's influent pipeline, ensuring alignment with real operational conditions. The system adopted an integrated AAO+MBR process (Supplementary Fig. 15b), comprising a regulating tank, anaerobic tank, anoxic tank, aerobic tank, and membrane tank (equipped with parallel intact MBR modules and CTDM modules). New PVDF hollow-fiber membranes (matching the original specifications of the plant's decommissioned units) as the control group.

CTDM Fabricated from 6-year-old waste MBR membranes (collected from the plant's decommissioned units). The CTDM system consisted of 5 independent modules, each containing 36 hollow-fiber membrane filaments (80 cm in length per filament). . For each filament, the PVDF separation layer was peeled off in equal proportion (via precision blade cutting and digital caliper area measurement) to achieve a uniform total support layer exposure area of 5% across all filaments and modules.

The system operated continuously under typical municipal wastewater treatment conditions:

- Hydraulic retention time: 12 h (anaerobic tank: 2 h, anoxic tank: 3 h, aerobic tank: 6 h, MBR tank: 1 h).
- Aerobic tank MLSS: 3300 ± 200 mg L^{-1}.
- Aeration intensity (aerobic tank): 2.5 m^3 ($m^{-2}\cdot h^{-1}$).
- Filtration flux (MBR/CTDM): 15 LMH.

To systematically track the structural stability of the membrane during operation, membrane samples were collected at three key time points (0 day, 20 days, and 45 days

of continuous operation) from the CTDM system. The collected samples were first photographed to record the macroscopic surface state (e.g., PVDF layer integrity, biofilm attachment), and then subjected to scanning electron microscopy (SEM) characterization to observe the microscopic structural changes of the support layer (e.g., pore size distribution, skeleton integrity) and the PVDF separation layer. This monitoring strategy ensures the timely capture of potential structural changes of the membrane during long-term operation, providing direct evidence for evaluating the stability of the partially peeled membrane.

3. Ultrasonic and NaClO Immersion Stability Tests

Short membrane filaments (1 cm in length, half with PVDF layer peeled off, half intact) were prepared as test samples, with three parallel samples for each treatment group. Ultrasonic treatment: Samples were placed in an ultrasonic cleaner (power: 100 W, frequency: 40 kHz) for cyclic oscillation treatment, with a cycle of 10 min on/10 min off, and total treatment duration of 48 hours. Sodium hypochlorite immersion treatment: Separate samples were immersed in a 15% sodium hypochlorite solution (consistent with the chemical cleaning concentration in the main experiment) for 48 hours at room temperature (22 ± 3℃), with gentle stirring every 12 hours to ensure uniform contact.

Before and after each treatment, the samples were photographed with a digital camera to observe the boundary integrity between the peeled and intact areas of the PVDF layer. For further microscopic verification, selected samples were subjected to scanning electron microscopy (SEM) characterization to evaluate the structural stability of the PVDF-support layer interface.

4. Computational Fluid Dynamics (CFD)

Computational Fluid Dynamics (CFD), one of the important technologies in the field of fluid mechanics in the 21st century, uses numerical methods to solve the governing equations of fluid mechanics on a computer to predict flow field behavior. The most basic consideration of CFD is to process continuous fluids in a discrete manner on a computer. The main steps of numerical flow field simulation include: grid generation for flow channels, setting of computational parameters, computational solution, and post-processing. The mainstream approach is the grid-based method, which discretizes the spatial region into small cells to form a three-dimensional grid or lattice, and then applies appropriate algorithms to solve the equations of motion (Navier-Stokes equations for viscous fluids). Additionally, such a grid can be irregular (e.g., composed of triangles in 2D and tetrahedrons in 3D) or regular. Under existing computational capabilities, turbulence models are typically used to simulate turbulent flows. The Baseline (BSL) k-ω model was used in this calculation, and its two equations are:

$$\frac{\partial(\rho k)}{\partial t}+\frac{\partial}{\partial x_j}(\rho U_j k)=\frac{\partial}{\partial x_j}\left[(\mu+\frac{\mu_t}{\sigma_{k3}})\frac{\partial k}{\partial x_j}\right]+P_k-\beta'\rho k\omega+P_{kb} \quad (1)$$

$$\frac{\partial(\rho\omega)}{\partial t}+\frac{\partial}{\partial x_j}(\rho U_j\omega)= \quad (2)$$

$$\frac{\partial}{\partial x_j}\left[(\mu+\frac{\mu_t}{\sigma_{\omega\varepsilon}})\frac{\partial\omega}{\partial x_j}\right]+(1-F_1)2\rho\frac{1}{\sigma_{\omega 2}\omega}\frac{\partial k}{\partial x_j}\frac{\partial\omega}{\partial x_j}+\alpha_3\frac{\omega}{k}P_k-\beta_3\rho\omega^2+P_{\omega b}$$

The values of each item are as shown in the Table S10 and S11.

The fluid is an incompressible fluid. The solid boundaries within the computational domain are treated as no-slip walls, and the boundaries around the water pool are of the Entrainment type.

5. Fouling model equations

Fouling model equations corresponding to different membrane fouling mechanisms under various operation modes are listed in Table S12. All model parameters were obtained by nonlinear data fitting using Origin software[1]. In these equations, *J* represents the real-time filtration flux (L m^{-2} h^{-1}); J_0 is the initial filtration flux at the start of filtration, with a value of 38 L m^{-2} h^{-1} in this study; t represents the filtration time (s). *Kb* is the complete blocking constant (0.00465 s^{-1}), which describes the membrane fouling behavior caused by particles completely sealing membrane surface pores. *Ki* is the intermediate blocking constant (2.03164×10^{-4} m^{-1}), characterizing the partial pore blockage and overlapping deposition of pollutants on the membrane surface. *Ks* is the standard blocking constant (3.16733×10^{-4} m^{-1}), reflecting the fouling process caused by pollutant particles depositing and narrowing the internal channels of membrane pores. *Kc* is the cake filtration constant (8.76932×10^{-6} m^{-2} s), which quantifies the flow resistance generated by the continuous accumulation and growth of the cake layer on the membrane surface.

6. xDLVO theory

In order to compute the physicochemical interaction occurring between the microbial bacteria present in sludge and the surface of the internal substrate layer and an external PVDF filtration membrane, the Derjaguin approximation (DA) approach was adopted. In this method, the bacterial cell was simplified to a spherical surface and treated as a series of concentric rings. Generally, it is calculated for the interaction between two smooth planes, and the total interaction energy is expressed as follows:

$$U^{TOT} = U^{LW} + U^{EL} + U^{AB} \tag{3}$$

where U^{TOT} represents the total interaction energy, U^{LW} represents the van der Waals free energy, U^{EL} represents the electrostatic double layer free energy, and U^{AB} represents the Lewis acid-base free energy (The data of U^{LW}, U^{EL}, and U^{AB} are presented in Supplementary Fig. 6). The three types of interactions between a smooth spherical surface and a smooth flat plate can be determined by using Eqs. (4) - (6):

$$U^{LW}(h) = 2\pi\Delta G_{h_0}^{LW}\frac{h_0^2 a_c}{h} \tag{4}$$

$$U^{EL}(h) = \pi\varepsilon_r\varepsilon_0 a_c\left[2\zeta_m\zeta_s \ln\left(\frac{1+e^{-kh}}{1-e^{-kh}}\right) + (\zeta_m^2 + \zeta_s^2)\ln(1 - e^{-2kh})\right] \tag{5}$$

$$U^{AB}(h) = 2\pi a_c\lambda\Delta G_{h_0}^{AB} exp\left(\frac{h_0-h}{\lambda}\right) \tag{6}$$

Here, h_0 represents the minimum separation distance between the microbial bacteria in sludge and the membrane, typically with a value of 0.158 nm. h denotes the separation distance between the microbial bacteria in sludge and the surface of the internal substrate layer and an external PVDF filtration membrane. $\Delta G_{h_0}^{LW}$ and $\Delta G_{h_0}^{AB}$ stand for the corresponding LW and AB energies at the minimum separation distance, expressed in mJ m^{-2} (This value was calculated using Equations (8) and (9) and shown in Supplementary Table S5). a_c represents the radius of the bacterial cell (Supplementary Table S4). $\varepsilon_r\varepsilon_o$ is the dielectric constant of the mixture with a value of 78.85 and 8.85×10^{-12} F m^{-1}. ζ_m and ζ_s are the Zeta potentials on the bacterial cell

and electrode surfaces, with the unit of mV. (Supplementary Table S4). λ indicates the characteristic attenuation length of acid - base action in water, and its typical value is around 0.6 nm. k represents the length of the inverse Debye shield, and the formula for calculating the inverse Debye length is given as:

$$k = \sqrt{\frac{e^2 \sum n_i z_i^2}{\varepsilon_r \varepsilon_0 kT}} \tag{7}$$

where e is the unit charge; n_i represents the number concentration of the ion; z_i indicates the valence state of the ion; *K* is the Boltzmann constant, usually taken as 1.38 × 10^{-23} J K^{-1}; *T* represents the absolute temperature of the environment. The typical value of k is around 3.28×108 m^{-1}.

The interaction energies per unit area between two infinite planes, $\Delta G_{h_0}^{LW}$ and $\Delta G_{h_0}^{AB}$, can be calculated from Eqs. (8) and (9):

$$\Delta G_{h_0}^{LW} = 2\left(\sqrt{\gamma_s^{LW} - \gamma_\omega^{LW}}\right)\left(\sqrt{\gamma_m^{LW} - \gamma_\omega^{LW}}\right) \tag{8}$$

$$\Delta G_{h_0}^{AB} = 2[\sqrt{\gamma_\omega^+}(\sqrt{\gamma_m^-} + \sqrt{\gamma_s^-} - \sqrt{\gamma_\omega^+}) + \sqrt{\gamma_\omega^-}(\sqrt{\gamma_m^+} + \sqrt{\gamma_s^+} - \sqrt{\gamma_\omega^+}) - (\sqrt{\gamma_m^- \gamma_s^+} + \sqrt{\gamma_m^+ \gamma_s^-})] \tag{9}$$

In these equations, γ_s^{LW}, γ_m^{LW} and γ_ω^{LW} represent the van der Waals action energy components of the surface tension among the membrane, microbial bacteria in sludge, and water, in units of mJ m^{-2}. γ^+ and γ^- denote the corresponding electron acceptor and electron donor tension components. The van der Waals action energy components of the membrane and microbial bacteria in sludge surface tension, as well as the corresponding electron donor and electron acceptor tension components, can be calculated using the known surface tension parameters γ_l^{LW}, γ^+ and γ^- on the membrane, the surface of the membrane forming the filter cake layer, and the host biofilm electrode. The contact angles of the three liquids on the surface of the membrane and the surface of the membrane forming the filter cake layer are calculated by the Lifshitz - van der Waals acid - base method and the extended Young's equation:

$$\gamma^{TOT} = \gamma^{LW} + \gamma^{AB} \tag{10}$$

$$\gamma^{AB} = 2\sqrt{\gamma^{+}\gamma^{-}} \tag{11}$$

$$\gamma_l^{TOT}(1 + \cos\theta) = 2\left(\sqrt{\gamma_s^{LW}\gamma_l^{LW}} + \sqrt{\gamma_l^{-}\gamma_s^{+}} + \sqrt{\gamma_l^{+}\gamma_s^{-}}\right) \tag{12}$$

where γ^{TOT} represents the total surface tension; γ^{LW} and γ^{AB} denote the surface energy components associated with van der Waals action and Lewis acid - base reaction, respectively; θ is the contact angle between a solid and a liquid (Supplementary Table S4). Subscripts s and l represent target solids and probe liquids (ultrapure water, glycerol, and n-hexadecane).

7. Questionnaire Survey on Market Share of Supported Curtain-Type MBR Membranes Among Major Manufacturers in China

Supplemented market share data via a professional questionnaire survey. A structured questionnaire was developed covering five core modules: (1) Basic enterprise information (to confirm industry representation); (2) Total MBR membrane production scale (to weight market share); (3) Product structure analysis (to clarify the proportion of curtain-type/hollow fiber membranes among total MBR products); (4) Key technical details of curtain-type membranes (focusing on the proportion of products with inner liner tube/central support tube); (5) Market application distribution (to verify consistency with large-scale MBR application scenarios).

Fifteen major MBR membrane manufacturers in China were selected as survey objects, covering different production scales (from 500,000 to >10 million m^2 $year^{-1}$) and geographical distributions (East, North, and South China), which were screened based on the 2024 China Membrane Industry Association Annual Report to ensure representativeness of the national market. Questionnaires were distributed via email and face-to-face interviews in November 2025, with two follow-ups for non-respondents to improve the response rate. Finally, 10 valid questionnaires were recovered, with an effective response rate of 66.7%.

For production scale data involving commercial confidentiality, the midpoint of the selected range was used for quantification (e.g., 3 million m^2 $year^{-1}$ for the range of 1-5 million m^2 $year^{-1}$) — a widely accepted method for confidential industrial data processing in LCA studies. The market share of supported hollow fiber membranes was calculated using weighted average based on production scale:

$$proportion = \Sigma\ (Production\ scale\ \times\ Proportion\ of\ curtain\ membranes\ \times\ Proportion\ of\ supported\ curtain\ membranes)\ /\ \Sigma\ Production\ scale \qquad (13)$$

The following section is the questionnaire survey：

Questionnaire on MBR Membrane Production and Market Status

Dear Expert/Director,

We are a research team from the School of Environment and Resources, Zhejiang A&F University. This survey aims to investigate the current status and future trends of China's MBR curtain-type membrane industry chain, focusing on collecting basic information on membrane materials, products, and market applications. All collected data will only be used for macro-industry analysis.

Part 1: Basic Enterprise Information

Enterprise Name: ______________________________

Address:______________________________________

Email: ______________________________________

Part 2: Total MBR Membrane Production Scale

Please provide accurate data if possible. If it is inconvenient to disclose exact figures, please check the range.:

□ < 500,000 m^2

□ 500,000 - 1,000,000 m^2

□ 1,000,000 - 5,000,000 m^2

□ 5,000,000 - 10,000,000 m^2

□ > 10,000,000 m^2

□ accurate data:____________

Part 3: Product Structure Analysis

1. Please estimate the output proportion of various MBR membrane products of your company (total 100%):

□ Flat-sheet Membrane:________________________________

□ Curtain-type Membrane:______________________________

□ Others (e.g., Spiral-wound):___________________________

2. Please estimate the output proportion of various MBR membrane material of your company (total 100%):

□ PVDF:____

□ PTFE:____

Part 4: Key Technical Details of Curtain-type Membrane

1. What is the output proportion of products with inner liner tube/central support tube among all curtain-type membranes produced by your company?

□ < 20%

□ 20% - 50%

□ 50% - 80%

□ > 80%

□ Almost all (>95%)

□ accurate data:________________

2. What are the main advantages of producing curtain-type membranes with inner liner tubes (multiple choices allowed)?

□ Enhanced Filament Strength

□ High-Intensity Cleaning Requirement

□ Extended Module Lifespan

□ Meeting Project Reliability Requirements

□ Others:____

Part 5: Market Application Distribution

Please estimate the main application fields and their proportions of MBR membrane products produced by your company (total 100%):

□ Municipal Wastewater:____

□ Industrial Wastewater:____

□ Agricultural Wastewater:____

□ Decentralized Rural Sewage:____

□ Others (e.g., Drinking Water, Seawater/Brackish Water Desalination, Reclaimed Water):____

Supplementary references

1. Hermia, J. Constant pressure blocking filtration laws-application to power-law non-Newtonian fluids. *Trans. Inst. Chem. Eng.* **60**, 183-187 (1982).
